# Anisakid biodiversity in two young harbour seals (*Phoca vitulina* L.) from coastal South-West Norway

**DOI:** 10.1007/s00436-025-08559-0

**Published:** 2025-09-26

**Authors:** Paolo Cipriani, Lucilla Giulietti, Marialetizia Palomba, Veronica Rodriguez Fernandez, Simonetta Mattiucci, Arne Bjørge, Arne Levsen, Miguel Bao

**Affiliations:** 1 Institute of Marine Research (IMR), Nordnes, Bergen Norway; 2 Dept. of Public Health and Infectious Diseases, Section of Parasitology, Sapienza University of Rome, Rome, Italy; 3 Dept. Ecological and Biological Sciences (DEB), Tuscia University, Viterbo, Italy; 4 Institute of Marine Research (IMR), Oslo, Norway

**Keywords:** *Phoca vitulina*, North-East Atlantic ocean, Pinnipeds, *Anisakis simplex (s.s.)*, *Phocanema*, *Contracaecum*

## Abstract

Anisakid nematodes are widespread marine parasites with complex life cycles involving invertebrates and fish as intermediate or transport hosts, and marine mammals as definitive hosts. Despite their ecological importance, and the zoonotic potential associated with the larval stages found in fish, recent data on anisakid species diversity in pinnipeds from Norwegian waters remain scarce. In this study, we investigated anisakid infections in two juvenile harbour seals (*Phoca vitulina*) stranded along the southern coast of Norway. Gastrointestinal nematodes were collected, morphologically classified to the genus level, and subsequently identified to species level through molecular analyses of mitochondrial (mtDNA *cox2*) and nuclear (rDNA ITS) markers.

Five anisakid species were identified: *Contracaecum osculatum* sp. A (reported here for the first time in harbour seals), *C. osculatum* (sensu stricto), *Phocanema decipiens* (s.s.), *P. krabbei*, and *Anisakis simplex* (s.s.). The latter species was found in unexpectedly high abundance and in fully developed adult stages in one of the seals. Notably, these adult *A. simplex* (s.s.) exhibited large body size, in contrast with previous studies reporting either absence or minimal presence of adults in harbour seals. The underlying mechanisms promoting growth and reproductive development of *A. simplex* (s.s.) in this host species remain unclear, but may involve a combination of host-specific physiological traits, environmental factors, and parasite phenotypic plasticity. Gross pathological examination revealed multiple gastric and intestinal ulcers in the same seal, including seven crateriform lesions consistent with ulcerative gastritis and enteritis, associated with nematode attachment and feeding. These findings expand the current knowledge on anisakid diversity in *P. vitulina* and provide novel evidence of its role as a definitive host for *A. simplex* (s.s.) in Norwegian coastal waters. Furthermore, the results suggest that competitive interactions among anisakid species, combined with ecological and physiological host factors, may facilitate the development and maturation of *A. simplex* (s.s.) in harbour seals. Further studies are warranted to assess the frequency and health implications of such infections in wild pinniped populations.

## Introduction

Anisakid nematodes (Nematoda, Anisakidae) of the genera *Anisakis*, *Phocanema*, *Phocascaris* and *Contracaecum* are marine parasites with a heteroxenous life cycle involving invertebrates as intermediate host, fish and cephalopods as paratenic transport hosts, and marine mammals or fish-eating birds as definite ones (only some *Contracaecum* species) (reviewed in Mattiucci and Nascetti 2008). Adult worms develop and reproduce in the stomach of marine mammals (definitive hosts), and eggs of the parasites are released into seawater with host feces. Within the eggs, the larvae undergo embryonation and develop into second and third-stage (L3) larvae (Køie et al. 1995), which are then ingested by crustaceans such as copepods or euphausiids. Fish or squid (paratenic/transport hosts) become infected by feeding on these infected crustaceans. Marine mammals acquire infection by consuming infected prey, allowing anisakid L3 larvae to develop into fourth-stage larvae (L4) and ultimately into adults. Some species are of considerable economic and public health importance due to the zoonotic potential of their infective L3 larvae. These larvae are frequently found in the musculature and visceral organs of fish and cephalopods, posing risks to human health, compromising seafood quality, and negatively impacting fisheries (EFSA 2024; reviewed in Mattiucci et al. 2018). Pinnipeds are known to host a wide range of anisakid nematodes, including several species that remain poorly studied (reviewed in Mattiucci and Nascetti 2008). Molecular studies conducted over the past two decades have expanded our understanding of the diversity of pinniped-infecting anisakids, helping to identify and resolve complexes of cryptic species within the genera *Phocanema*, *Contracaecum*, and *Phocascaris* (Mattiucci and Nascetti 2008; Nascetti et al. 1993; Paggi et al. 1991). Notably, the genus *Phocanema* Myers, 1959, has recently been resurrected to accommodate the species formerly assigned to the *Pseudoterranova decipiens* (s.l.) complex that mature in pinnipeds, including *P. decipiens* (s.s.), *P. krabbei, P. bulbosum*, *P. azarasi*, and *P. cattani*, maturing in pinnipeds, while the genus *Pseudoterranova* remains valid only for two species infecting kogiid whales (Bao et al. 2023).

Genetic studies have shown that each species within these genera exhibits varying degree of specificity towards different pinniped definitive host species, with host-parasite interactions influencing anisakid fitness (Mattiucci and Nascetti 2008; Nascetti et al. 1986, 1993; Paggi et al. 1991). Despite the extensive literature on anisakids, updated studies on their epidemiology, species identification, and host-specificity in seal populations from NE Atlantic waters remain limited.

In North-East (NE) Atlantic and Arctic waters, the harbour seal *Phoca vitulina* Linnaeus, 1758, is a common definitive host for several anisakid species (Mattiucci and Nascetti 2008). These include anisakids from the genera *Phocanema* (formerly *Pseudoterranova*), commonly referred to as the seal or cod worm; *Phocascaris, Contracaecum,* and *Anisakis*, also referred to as the whale or herring worm (Ólafsdóttir and Hauksson 1997; Young 1972). A recent study investigated 13 harbour seals in the Kattegat- North Sea transition zone (Kumas et al. 2025), reporting a wide diversity of anisakid nematodes, including *P. krabbei*, *P. decipiens* (s.s.), *C. osculatum* (s.s.) and *A. simplex* (s.s.). Other studies on anisakids based on larger samples of harbour seals have been conducted in pinniped populations from the Kattegat, Skagerrak, and Baltic waters (Lunneryd 1991), as well as in the German Wadden Sea (Lehnert et al. 2007; Siebert et al. 2007). In Norwegian waters, the last comprehensive investigations were conducted over three decades ago, during which seal worm infections were documented in harbour seals from the outer Oslofjord (Aspholm et al. 1995; Ólafsdóttir and Hauksson 1997). An earlier study by Bjørge (1987) examined 127 harbour seals along the Norwegian coast, providing valuable baseline parasitological data. However, the most of these studies precedes development of molecular techniques, which now facilitate the discrimination of cryptic anisakid species.

The harbour seal is a member of the Phocidae family which comprises 19 species of true seals. In the NE Atlantic, six species of true seals are found, but only the harbour seal and the grey seal, *Halichoerus grypus* (Fabricius, 1791), inhabit the Norwegian mainland coast throughout the year, while the bearded seal, *Erignathus barbatus* (Erxleben, 1777), may occur sporadically in coastal Finnmark. Harbour seals are distributed along the entire Norwegian coastline, favouring shallow coastal waters. They use three distinct habitat types for resting and pupping: coastal skerries and shallow islets, deep fjords, and estuarine sandbanks (Bjørge 1991). Harbour seals typically remain in proximity to their haul-out areas throughout the year (Bjørge 1987). Along the southern and southeastern Norwegian coasts, seal skerries remain exposed throughout the tidal cycle, while on the western and northern coasts, many skerries are only visible at low tide. Adult harbour seals exhibit an opportunistic feeding strategy, preying on a diverse range of fish, cephalopods, and crustaceans (Härkönen 1987; Olsen and Bjørge 1994). Consequently, the highest densities of harbour seals are found in Sør-Trøndelag and Nordland counties, where suitable haul-out habitats and abundant prey coincide.

In recent years, under the increasing impact of human activities, harbour seal populations have been exposed to multiple stressors—including reduced haul-out opportunities, shifts in prey availability and competition, and expanding pathogen ranges—that are profoundly affecting their health and parasite dynamics (Blanchet et al. 2021). Along the Norwegian coast, harbour seal populations have fluctuated due to severe outbreaks of infectious diseases. Notably, repeated epizootics of Phocine Distemper Virus (PDV) have caused substantial regional declines (Markussen 1992), as has Seal Influenza A(H10N7) virus (Bodewes et al. 2015). The most recent surveys estimate that the total mainland population has fluctuated around 7,000 individuals over the past two decades (Nilssen and Bjørge 2009; Nilssen et al. 2023).

The present study investigated the characteristics of anisakid nematode infections in two harbour seals stranded along the Norwegian northern North Sea coast, providing new insights into the species composition and ecology of these parasites in this definitive host.

## Materials and methods

**Fig. 1:**
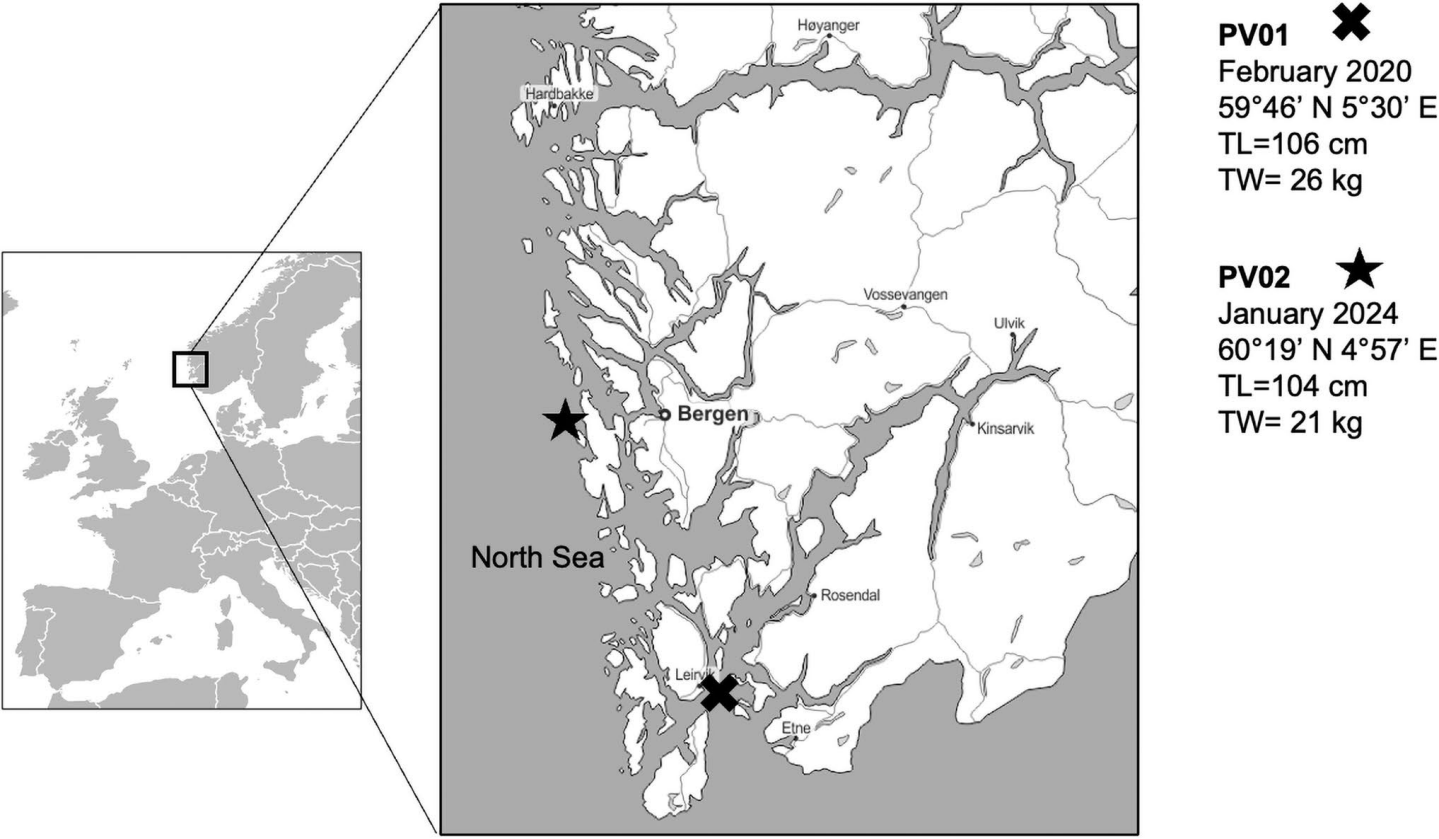
Map showing the stranding locations of two harbour seals (*Phoca vitulina*), PV01 (marked with an “X”) and PV02 (marked with a star), examined in this study. Associated biometric data include date, coordinates, total length (TL), and total weight (TW)

Two young harbour seals (one male hereby indicated as PV01, and one female hereby indicated as PV02) were found drowned, after becoming accidentally tangled in fishing nets along the coast near Bergen, in western Norway (Fig. 1). Recovery locations and dates, total length and weight, as well as sex, are shown in Fig. 1. Based on size and weight, both seals were estimated to be approximately one year of age (Markussen et al. 1989). The animals were processed within 24 h of death. Stomach tissue, contents, and intestines were examined for the presence of nematodes and associated lesions. The presence or absence of ulcerative lesions in the mucosa was recorded and photographed. Nematodes attached to the stomach wall and in the digestive tract were counted and collected. All recovered nematode specimens were washed in physiological saline and classified into developmental stages as follows: L3 (labia absent, boring tooth present), L4 (labia present, boring tooth absent, sex not ascertained), adult males (labia present, caudal papillae present, spicules present), immature adult females (labia present, eggs absent) and mature adult females (labia present, eggs present) (Grabda 1976; Herreras et al. 2004). Genus identification was conducted using light microscopy, based on the morphology of the head, digestive tract and tail regions of nematodes mounted on glass slides in physiological saline. Diagnostic features include cephalic structure, ventriculus shape and length, presence/absence of caeca and/or ventricular appendix, tail morphology, and cuticle ornamentation (Berland 1961, 1963; Davey 1971; Mattiucci and Nascetti 2008; McClelland 1980). Remaining body fragments were retained in 70% ethanol for subsequent molecular analysis.

### Molecular identification of anisakid parasites

A total of 54 nematode specimens were identified by sequence analysis of the mitochondrial cytochrome c oxidase II (mtDNA *cox*2) gene and the ITS region of rDNA. DNA extraction was carried out using DNeasy Blood & Tissue kits (Qiagen), following the manufacturer's guidelines. Amplification of the *cox*2 gene was done using the primers 211F (5′-TTTTCTAGTTATATAGATTGRTTTYAT-3′) and 210R (5′-CACCAACTCTTAAAATTATC-3′) (Nadler and Hudspeth 2000). The polymerase chain reaction (PCR) was conducted as described by Mattiucci et al. (2014). The ITS region rDNA, including the first internal transcribed spacer (ITS-1), the 5.8S and the second transcribed spacer (ITS-2), was sequenced using the primers NC5 (forward; 5′-GTA GGT GAA CCT GCG GAA GGA TCA TT-3′) and NC2 (reverse; 5′-TTA GTT TCT TTT CCT CCG CT-3′) (Zhu et al. 2000). PCR was carried out according to the procedures reported in Zhu et al. (2000). Purification and sequencing of PCR products were performed by Eurofins (Cologne, Germany).

Sequence alignments were performed using ClustalX v2.0 (Larkin et al. 2007) and subsequently edited and trimmed manually with BioEdit v7.0.5.3 (Hall 1999). The resulting sequences were then analyzed and compared against the GenBank database using the BLAST algorithm (https://www.ncbi.nlm.nih.gov/BLAST).

## Results

A total of 1168 nematodes were collected from the stomach of the two seals. Out of these, 986 showed morphological characters consistent with *Anisakis* (s.l.), including a long and curved ventriculus; 49 showed features consistent with *Phocanema* (s.l.), characterized by the presence of an intestinal caecum running along the ventriculus; and 95 displayed traits typical of *Contracaecum* (s.l.), including an intestinal caecum, ventriculus, and ventricular appendix. The number of larval and adult specimens assigned to each genus collected from the two seals are reported in Table 1.

**Table 1:** Developmental stage and species composition of anisakid nematodes recovered from the stomach of the two juvenile harbour seals (PV01 and PV02) stranded along the southwestern coast of Norway. Parasites were morphologically assigned to *Anisakis simplex* (s.l.), *Phocanema* spp., and *Contracaecum* spp., and classified into third-stage larvae (L3), fourth-stage larvae (L4), and adult stages

		*Anisakis simplex* (s.l)	*Phocanema* spp.	*Contracaecum* spp.
PV01	L3	19	0	4
	L4	11	0	5
	Adult	4	7	5
	Total (%)	34 (61.8%)	7 (12.7%)	14 (25.5%)
PV02	L3	530	42	0
	L4	279	0	81
	Adult	181	0	0
	Total (%)	990 (89.0%)	42 (3.8%)	81 (7.2%)

**Fig. 2:**
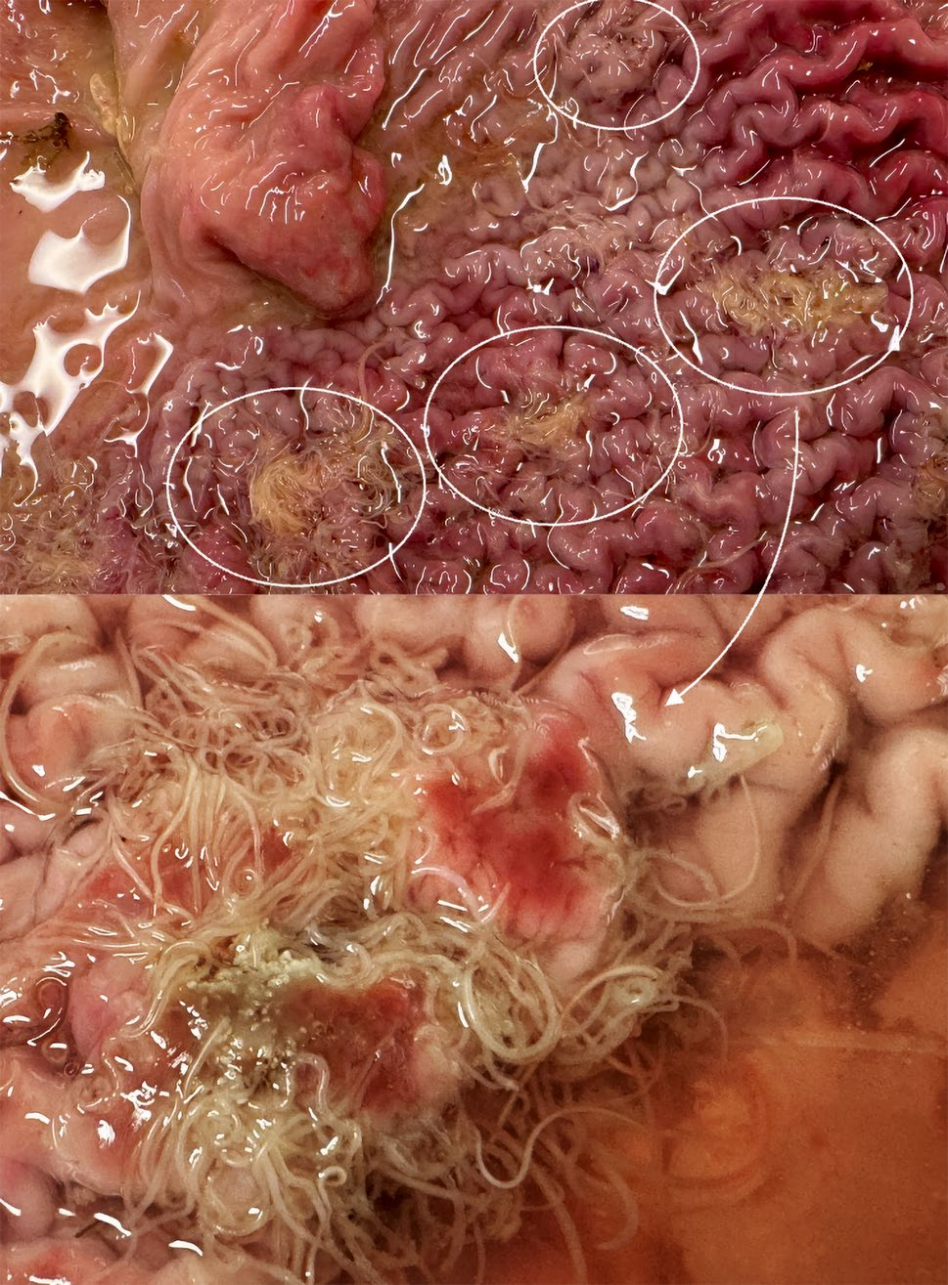
Ulcerative lesions in the stomach wall of harbour seal PV02, associated with nematodes clusters. The lower panel shows a close-up of one lesion
  *A. simplex* (s.l.) was the most abundant nematode, and a total of 181 mature males and females were found (Fig. 3), with a mean length of 60 mm (Table 1). The sex ratio of adult *A. simplex* (s.l.) was 59% females and 41% males.

The nematode burden, developmental stages, and associated gastric pathology observed in each of the two harbour seals (PV01 and PV02) were as follows:

- *PV01*
  Digested fish in the stomach. A total of 55 nematodes were counted, no ulcers observed, but several *Anisakis* (s.l.) specimens were deeply embedded in the mucosa of the stomach wall. Only 4 of the 34 *Anisakis* (s.l.) specimens were adults, with a mean body length of 43 mm, whereas the remaining individuals were in the L3 or L4 larval stages (Table 1). All 7 *Phocanema* (s.l.) were adults with 34 mm mean body length, while 5 of 14 *Contracaecum* (s.l.) were L4 stage larvae and 5 were adults, with mean body length 32 mm (Table 1). The sex ratios were 75% females and 25% males for adult *Anisakis* (s.l.), 85% females and 15% males for *Phocanema* (s.l.), and 80% females and 20% males for adult *Contracaecum* (s.l.).
- *PV02*:
  A total of 1113 nematodes were counted. Over 700 worms were found free in the stomach lumen, mostly within partially digested fish. The remaining specimens, nearly 1/3 of all collected nematodes, were attached to the gastric mucosa, sometimes concentrated within ulcerative lesions that appeared as crater-like wounds (Fig. 2). These ulcers frequently contained dense aggregations of nematodes, with their tails protruding into the lumen. In two of such lesions, clusters of nematodes -initially identified as *Anisakis simplex* (s.l.) at various stages of development- were firmly anchored at the centre of each ulcer (Fig. 2).

**Fig. 3:**
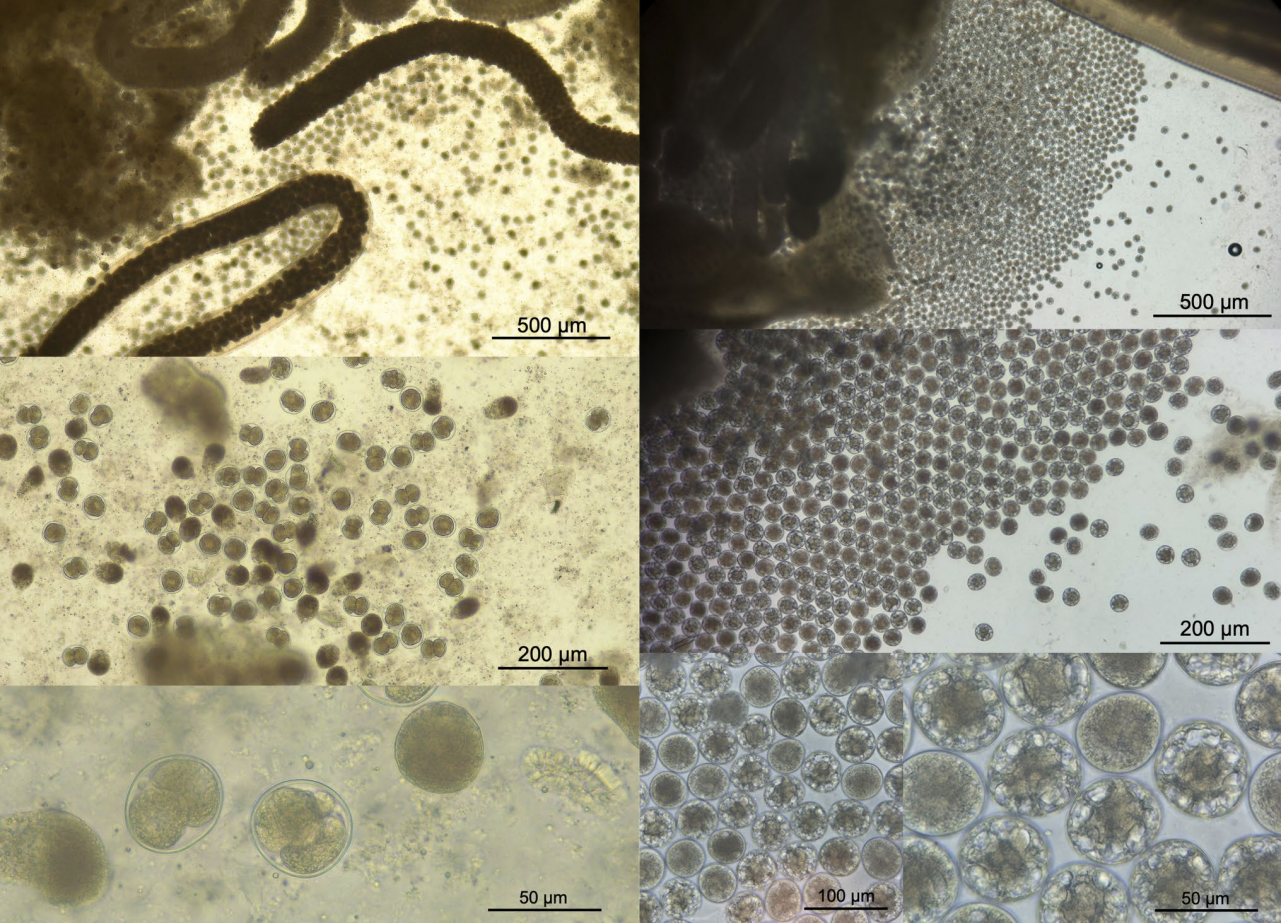
Uterus of adult *Anisakis simplex* (s.s.) containing numerous developed eggs. The upper panel shows a low magnification overview of the reproductive system, while the lower panel displays a higher magnification image of the same structure, revealing clearly defined eggs with visible larvae inside the eggshells
  All the 42 *Phocanema* (s.l.) specimens found were at L3 stage (ranging 13 to 25 mm in body length), while the 84 *Contracaecum* (s.l.) counted were all L4 (mean length 21 mm, ranging 11 to 27 mm), i.e. no fully mature specimens were observed (Table 1).

### Molecular identification of anisakid nematodes

The partial *cox*2 mtDNA sequence (580 bp) obtained from 21 specimens with *A. simplex* (s.l.) morphological features showed 99.8% identity with *A. simplex* (s.s.) (GenBank accession number, a.n.: OR568602). The 21 rDNA ITS sequences obtained from the same specimens were 100% identical to each other and showed 100% identity with *A. simplex* (s.s.) sequences in GenBank (a.n.: MT250916). Among these, the chromatogram from a mature male worm (total length: 55 mm) exhibited overlapping double peaks at two diagnostic positions—position 278 (C in *A. pegreffii*, T in *A. simplex* (s.s.)) and position 294 (C in *A. pegreffii*, T in *A. simplex* (s.s.))—as defined by D’Amelio et al. (2000), suggesting a heterozygous pattern. Additionally, the chromatogram from a mature female worm (also 55 mm in length) displayed a double peak at position 278, further indicating possible heterozygosity at this diagnostic site.

Out of the 18 mtDNA *cox*2 sequences obtained from nematode specimens displaying *C. osculatum* (s.l.) morphological features, 17 showed 99.5% similarity with *C. osculatum* (s.s.) (a.n.: MT259584), while 1 worm showed 98.1% similarity with *C. osculatum* sp. A (a.n.: EU477203). Similarly, among the 10 rDNA ITS sequences obtained from a subsample of *Contracaecum* (s.l.) specimens, 9 were 100% identical and matched *C. osculatum* (s.s.) (a.n.: MN428820), while 1 specimen matched 100% with *C. osculatum* sp. A (a.n.: AB277825).

According to mtDNA *cox*2 sequence analyses, the 15 nematodes with *P. decipiens* (s.l.) morphological traits belonged to two species: 9 showed > 99% similarity with *P. decipiens* (s.s.) (a.n.: OP418116), while the remaining 6 matched > 99% % with *P. krabbei* (a.n.: OP418118).

Regarding a subsample of 9 individuals assigned to the species of *P. decipiens* (s.s.)—based on mtDNA *cox*2 sequences analysis- when sequenced at rDNA ITS, 2 of them showed 100% identity with *P. decipiens* (s.s.) (a.n.: JQ673263); while the remaining 7 showed 100% identity with the ITS deposited in GenBank for *P. krabbei* (a.n.: OP355454).

The majority of the *cox*2 mtDNA and rDNA ITS sequences obtained from the analyzed specimens were consistent in species identification, with the exception of one *Phocanema* (s.l.) individual, whose ITS sequence matched *P. krabbei*, while the *cox*2 sequence matched *P. decipiens* (s.s.).

Sequences of mtDNA *co*x2 and rDNA ITS here obtained were deposited in GenBank under the following accession numbers, organized by species and genetic marker: *Anisakis simplex* (s.s.) – *cox*2: PX290001–PX290003, PX290015–PX290016; ITS: PX285992. *Phocanema decipiens* (s.s.) – *cox*2: PX289998–PX290000, PX290013–PX290014; ITS: PX285993. *Phocanema krabbei* – *cox*2: PX290011–PX290012; ITS: PX285994. *Contracaecum osculatum* (s.s.) – *cox*2: PX290004–PX290007, PX290009–PX290010; ITS: PX285990. *C. osculatum* sp. A – *cox*2: PX290008; ITS: PX285991.

## Discussion

The present study revealed a high diversity of anisakid parasite species in two juvenile harbour seals sampled along the southwestern coast of Norway. The use of two widely applied genetic markers—mitochondrial *cox2* and the nuclear rDNA ITS region—enabled the identification of five species: *C. osculatum* sp. A (reported here for the first time in harbour seals), *C. osculatum* (s.s.), two *Phocanema* species (*P. decipiens* (s.s.) and *P. krabbei*), and *A. simplex* (s.s.). While most specimens were consistently identified across markers, some discordant results in species assignment were observed. Specifically, two nematodes identified as *A. simplex* (s.s.) based on *cox2* sequences exhibited heterozygous patterns in the ITS chromatograms at the two diagnostic SNP positions (278 and 294) that are typically used to distinguish *A. simplex* (s.s.) from *A. pegreffii* (D’Amelio et al. 2000). One adult male displayed double peaks at both positions, while an adult female showed a double peak at position 278 only. This ITS pattern has previously been reported in both *A. simplex* (s.s.) and *A. pegreffii* (Cavallero et al. 2014; Mattiucci et al. 2016), perhaps due to incomplete lineage sorting of ancestral polymorphism or a historical introgression event at this marker, leading to a shared polymorphism between the two species in the ITS-1 region of rDNA (Mattiucci et al. 2016). Similarly, a discordance between the assignments based on mtDNA *cox*2 sequence analysis and the rDNA ITS region was observed among the *Phocanema* specimens examined in this study. One individual showed an ITS sequence matching *P. krabbei*, while the *cox*2 sequence matched *P. decipiens* (s.s.). Mito-nuclear discordance has previously been documented among other anisakid species, such as L3-stage larvae of *A. simplex* (s.l.) in the Mediterranean Sea (Mladineo et al. 2017; Mattiucci et al. 2025). These discordance between mitochondrial and nuclear markers, as well the polymorphism observed in ITS, should be further investigated using additional nuclear loci with mendelian inheritance in these species, as recently applied in the evidence of mito-nuclear discordance between *A. pegreffii* and *A. simplex* (s.s.) in the Mediterranean Sea (Mattiucci et al. 2025).

Studies on anisakid nematodes in pinnipeds from the NE Atlantic remain scarce, particularly in northern and Arctic regions (Johansen et al. 2010), and most rely solely on morphological identification, thereby overlooking part of the biodiversity within these species. Most research to date has focused on other areas, such as the Baltic Sea, Wadden Sea, Icelandic waters, and the North Pacific (Brattey and Stenson 1993; Gabel et al. 2021; Karpiej et al. 2014; Kumas et al. 2025; Kuzmina et al. 2014; Lehnert et al. 2007; Marcogliese 2001; Ólafsdóttir and Hauksson 1997; Siebert et al. 2007; Skrzypczak et al. 2014; Templeman 1990; Walden et al. 2020; Young 1972; Zuo et al. 2018). As a result, the prevalence, diversity, and ecological role of anisakids in pinnipeds from the NE Atlantic remain poorly understood. In the North Sea region, a recent study investigated 13 harbour seals from Danish waters in the Kattegat–North Sea transition zone (Kumas et al. 2025), reporting an infection pattern comparable to the findings reported here, both in terms of species diversity and infection levels. In Norwegian waters, data on anisakid infections in harbour seals are outdated. The most recent investigations date back more than thirty years, when *C. osculatum* (s.l.) was morphologically identified in harbour seals from the outer Oslofjord (Aspholm et al. 1995). Bjørge (1987) similarly reported anisakid infections in 127 harbour seals along the Norwegian coast. The present study adds to these earlier works by providing the first genetic identification of *C. osculatum* sp. A in harbour seals, and by documenting a consistent infection with adult specimens of *A. simplex* (s.s.), a rare and only recently reported occurrence in this pinniped species (Kumas et al. 2025). Lehnert et al. (2007) and Siebert et al. (2007), in two large studies, investigated parasites and pathological findings in more than 400 harbour seals from the Wadden Sea, reporting infections with *P. decipiens* (s.l.) and *C. osculatum* (s.l.), but not detecting the presence of *A. simplex* (s.l.).

In recent years, genetic identification techniques have clarified that the *C. osculatum* (s.l.) complex comprises at least three sibling species: *C. osculatum* (s.s.), *C. osculatum* sp. A, and *C. osculatum* sp. B (reviewed in Mattiucci and Nascetti 2008; Mattiucci et al. 2017). Among the arctic members of the *C. osculatum* (s. l) complex, *C. osculatum* (s.s.) is commonly reported in seals and paratenic/transport fish hosts, and rarely in crustaceans such as amphipods and copepods, from the Baltic Sea, Skagerrak, and southern North Sea, with a generally decreasing prevalence at higher latitudes (Gabel et al. 2021; Køye and Fagerholm 1995; Kumas et al. 2025; Mattiucci and Nascetti 2008; Mattiucci et al. 2017; Pawlak et al. 2019; Smith and Wootten 1978; Zuo et al. 2018). It has also been found in ringed seals (*Pusa hispida*) from the North-West (NW) Atlantic (Nunavut, Canada) (Karpiej et al. 2014). However, in earlier studies, *C. osculatum* was commonly reported without discriminating between sibling species (e.g., Aspholm 1991; Jensen 2009; Ólafsdóttir and Hauksson 1997; Templeman 1990; Young 1972), or consistent identification of developmental stages (Skrzypczak et al. 2014). As a consequence, the exact species identity of the *C. osculatum* specimens reported in those earlier studies remains uncertain. In the present study, 95 *Contracaecum* (s.l.) specimens were recovered from the two harbour seals, with most genetically identified as *C. osculatum* (s.s.). The majority were L4, with only five individuals showing adult characteristics. These findings suggest that the North Sea, up to 62° N, remains within the geographical distribution range of *C. osculatum* (s.s.), likely offering suitable ecological conditions, including appropriate temperature, salinity, and host availability, for completing its life cycle. In addition, a single larva was identified as *C. osculatum* sp. A, representing the first genetic record of this species in harbour seals. However, it is likely that this species has been present in this host previously but remained unrecognized, as earlier studies—without molecular diagnostic tools—classified it under the broad taxon *C. osculatum* (s.l.). Genetic identification has previously confirmed *C. osculatum* sp. A in grey seals, ringed seals and bearded seals (*Erignathus barbatus*) from NE and NW Atlantic waters, as well as the NW Pacific off Japan (Karpiej et al. 2014; Mattiucci et al. 1998, 2017; Mattiucci and Nascetti 2008). Interestingly, in the present study, only a single L4 of *C. osculatum* sp. A was recovered in one of the harbour seals. Previous evidence indicates a stronger host association of *C. osculatum* sp. A with other phocid species, and a more arctic distribution both in definitive and paratenic/transport hosts (Levsen et al. 2022; Mattiucci and Nascetti 2008; Mattiucci et al. 2017).

The genus *Phocanema* remains the dominant anisakid genus found in adult harbour seals from the NE Atlantic (Aspholm 1991; Aspholm et al. 1995; Bjørge 1987; Kumas et al. 2025; Lehnert et al. 2007; Lunneryd 1991; Mattiucci and Nascetti 2008; Mattiucci et al. 2017; Ólafsdóttir and Hauksson 1998; Siebert et al. 2007). In the present study, two *Phocanema* species were identified, i.e. *P. decipiens* (s.s.) and *P. krabbei*. In PV01, only five adults *Phocanema* were found (mean length: 33.7 mm), while in PV02, they were all L3 (13–25 mm). Both these L3 and adult *Phocanema* specimens matched or resulted smaller than L3 typically observed in gadoid fishes from the same area, where *Phocanema* larvae can reach 40 mm in length (personal observation). This size discrepancy suggests that the present seals acquired these L3 parasites relatively recently, most likely feeding on infected intermediate or paratenic hosts, such as benthic crustaceans or small benthic fish.

To better understand the observed infection patterns, it is useful to briefly consider the life cycle of *Phocanema* (s.l.). This group of parasites genus uses benthic invertebrates as first intermediate host and fish as second intermediate or paratenic host. Final maturation occurs in the stomach of pinnipeds, which act as definitive hosts (McClelland 1990).

In the two juvenile harbour seals analysed in this study, *P. decipiens* (s.s.) and *P. krabbei* were detected in low numbers. High burdens of these parasite species have often been reported in both seals and fish hosts in proximity to areas hosting large seal colonies and haul-out sites (Bjørge 1987; Brattey et al. 1990; Jensen and Idås, 1992; Marcogliese and McClelland 1994). Along the southwestern coast of Norway, particularly in the region where these animals stranded, harbour seals are typically found in small, scattered coastal groups rather than in large, dense colonies. This may suggest that these juvenile harbour seals feed mostly on local coastal pelagic and demersal fish species, which often harbour *A. simplex* in high abundance*,* but only rarely carry *Phocanema* and *Contracaecum* spp. (Cipriani et al. 2025; Levsen et al. 2018; Lunneryd 1991).

*Anisakis simplex* (s.s.) is primarily a parasite of cetaceans, particularly toothed whales (e.g. porpoises, dolphins and orcas) and baleen whales (e.g. minke whale), which are widely distributed in Norwegian waters (Anderson 2006; Berland 2006; Cipriani et al. 2022; Lakemeyer et al. 2020; Lehnert et al. 2005; Ryeng et al. 2022; Siebert et al. 2006; Ugland et al. 2004). Several studies on anisakid infections in pinnipeds, including harbour seals, predominantly reported larval stages of this species. Adult specimens, or not specified developmental stages, were less frequent and not exceeding 40 mm in length when measured (Aspholm 1991; Jensen 2009; Kuzmina et al. 2014, 2018; Hansen and Malmstrøm, 2006; Lunneryd 1991; Mansfield and Beck 1977; Ólafsdóttir and Hauksson 1998; Scott and Fisher 1958; Skrzypczak et al. 2014; Walden et al. 2020; Young 1972). Kumas et al. (2025) recently reported the presence of sexually mature *A. simplex* (s.s.) in several harbour seals from Danish waters, with individuals carrying fully developed eggs. The authors concluded that this parasite, commonly known as the “whale worm,” is then capable of developing and reproducing in this seal species. The size of adult *A. simplex* varies depending on the definitive host species and size, reflecting this nematode’s phenotypic adaptability (Ugland et al. 2004). This may serve as a measure of the reproductive potential and, thus, the fitness of the parasite, as larger individuals tend to produce a higher number of fertile eggs which increases reproductive success and potential survival rates (Ugland et al. 2004). For instance, Ugland et al. (2004) reported a mean length of 106 mm for males and 126 mm for females of *A. simplex* in baleen whales, compared to 68 mm for males and 73 mm for females in dolphinids. In the present study, one of the seals (PV02) was heavily infected with *A. simplex* (s.s.), with several adult specimens showing mean length of 59.1 mm (55–69 mm) for males and 62.7 mm (50–75 mm) for females, values comparable to those reported in dolphinids from the same area (Ugland et al. 2004). These adult worms included large females containing embryonated eggs (Fig. 3) and males with fully developed spicules. In contrast, the other seal (PV01) exhibited size and relative proportions of *A. simplex* larval and adult stages consistent with previous findings in harbour seals (Kumas et al. 2025), with a mean length of 43 mm and females containing ripening eggs. Kumas et al. (2025) estimated that individual female Anisakis in harbour seal may produce nearly 30,000 eggs per day, but did not report the mean body length of the worms, thereby limiting any fitness-related correlation with parasite size.

The underlying reasons for the increased fitness of *A. simplex* (s.s.) in this particular seal host species remain unclear. Aside from the recent study by Kumas et al. (2025), which reported mature *A. simplex* (s.s.) at high prevalence and intensity in harbour seals, previous investigations—including those with comparable host sample sizes—reported lower abundances of *A. simplex* (s.l.), or shorter mean body lengths, even in the rare cases where adult specimens were observed (Aspholm 1991; Jensen 2009; Johansen 2012; Kumas et al. 2025; Scott and Fisher 1958). In harbour seals from Canadian Atlantic waters, adult *Anisakis* sp. were only rarely observed (Scott and Fisher; 1958). In two studies targeting harbour seal from the Oslofjord area, Aspholm (1991) found 49 adult *A. simplex* (s.l.) in 29 seals, while Jensen (2009) reported that all adult female *A. simplex* (s.l.) found in seven seals contained eggs in their uteri. Among 95 harbour seals from Icelandic waters, none harboured sexually mature *A. simplex* (s.l.) (Ólafsdóttir and Hauksson 1998). In coastal areas of the NW Atlantic, Brattey and Stenson (1993) found that 32% of 47 (15/47) investigated harbour seals from Newfoundland and Labrador were infected with *A. simplex*, although none were sexually mature. Moreover, no adult *A. simplex* (s.s.) were detected in harbour seals from the Baltic Sea or British waters (Skrzypczak et al. 2014; Young 1972). The atypical growth and development of *A. simplex* (s.s.) reported in this study, rarely observed in harbour seals before, raises questions regarding host specificity and preference, as well as interspecific competition, among anisakid nematodes in pinniped hosts, and pinniped host immune response. Indeed, the present findings, in accordance with the results recently reported by Kumas et al. (2025), indicate that under certain conditions, *A. simplex* (s.s.) can achieve high fitness and reproductive success in this host. The main challenge will be to determine whether these observations represent a previously overlooked behaviour of *A. simplex* (s.s.), or rather an opportunistic trend driven by the rapidly changing environmental conditions of the Anthropocene, where human-induced stressors such as climate change, overfishing, and habitat alteration increasingly alter marine ecosystems. As observed in a comprehensive meta-analysis by Fiorenza et al. (2020), different anisakid genera may exhibit varying resilience to global change. This analysis indicates that *Anisakis* spp. have globally increased over time, whereas *Phocanema* spp. have remained largely unchanged (Fiorenza et al. 2020). *Phocanema* spp. larvae are typically associated with coastal demersal habitats (McClelland 2002; Measures 2014), while most *Anisakis* spp. are linked to pelagic or mesopelagic realms (Mattiucci et al. 2018; Measures 2014; Smith and Wootten 1978). Coastal anthropogenic stressors—such as chemical contamination, nutrient enrichment, coastal development, and overfishing of coastal species—are therefore likely to impact *Phocanema* spp. life cycles more strongly than those of *Anisakis* spp. (Fiorenza et al. 2020).

Notably, the grey seal has also been identified as a potential definitive host for *A. simplex* (s.s.), as demonstrated by the presence of small numbers of adult worms (Brattey and Stenson 1993; Ólafsdóttir and Hauksson 1997; Young 1972). The occurrence of adult *Anisakis* has also been reported in other pinniped species, including harp seal (*Pagophilus groenlandicus*), ringed seal, bearded seal, spotted seal (*Phoca largha*), ribbon seal (*Histriophoca fasciata*) and northern fur seal (*Callorhinus ursinus*) (Brattey and Ni 1992; Kuzmina et al. 2014; Walden et al. 2020). Interestingly, the sibling species *Anisakis pegreffii* was described from the Mediterranean monk seal (*Monachus monachus*) (Campana-Rouget and Biocca 1955). Given these findings, *A. simplex* (s.s.) may preferably utilise cetaceans as definitive hosts while utilising pinnipeds as alternative definitive hosts, likely with a reduced fitness. The relatively low degree of host specificity, combined with the plasticity and adaptability of *A. simplex* (s.s.) across different life stages – ranging from crustacean intermediate hosts, fish and cephalopod second intermediate or paratenic hosts, and ultimately cetaceans or pinnipeds as definitive hosts – likely contributes to its status as one of the most prevalent parasitic helminths in North Atlantic waters (Aspholm et al. 1995; Gay et al. 2018; Jensen and Idås, 1992; Levsen et al. 2018; Mattiucci et al. 2017, 2018).

The two seals exhibited different infection levels, with one individual (PV02) showing a tenfold higher parasite intensity than the other. The cause of this difference remains unclear, as both seals were of similar age, originated from the same area, and were in good nutritional condition. Fish bone remains were detected in both stomachs, indicating recent feeding, which was further supported by the presence of developing larval stages of several parasite species. The more heavily parasitized seal exhibited gastric ulcers identified as crater-like lesions associated with dense aggregations of nematodes (Fig. 2). Specifically, seven ulcers were observed, each with clusters of worms protruding from the central area and firmly attached to the stomach wall (Fig. 2). These clusters accounted for approximately one third of the total nematodes counted, and were mostly composed of *A. simplex* (s.s.) at various developmental stages. According to their weight–length ratios, the seals appeared to be in good nutritional condition. Seals, like most marine mammals, typically show low to moderate gastric tissue reactions to anisakid nematode infections, commonly manifested as ulcerative and granulomatous gastritis, as well as eosinophilic, granulomatous, and catarrhal-lymphocytic enteritis (Lehnert et al. 2007; Siebert et al. 2007; Ten Doeschate et al. 2017). Elevated infection levels of gastric nematodes are often linked to weakened immune systems and may represent a consequence rather than a direct cause of declining health and rarely causing mortality in pinnipeds (Geraci and Aubin 1987; Hrabar et al. 2021; Lunneryd 1991; Ten Doeschate et al. 2017). The extent, incidence, and parasite species generally associated with these lesions remain unclear. In the Wadden Sea, only a single ulcer was reported in the stomach of a harbour seal infected with *P. decipiens* (s.l.) and *C. osculatum* (s.l.) out of 71 animals examined (Lehnert et al. 2007). Similarly, Siebert et al. (2007) observed rare cases of ulcerative gastritis in a large sample of 355 harbour seals from the same area, also infected with *P. decipiens* (s.l.) and *C. osculatum* (s.l.). By contrast, gastric lesions were reported in a large number of seals from the Kattegat and Skagerrak, infected with *P. decipiens* (s.l.), *C. osculatum* (s.l.), and *Anisakis simplex* (s.l.), most commonly one ulcer per seal, with up to six ulcers observed in a single individual (Lunneryd 1991). McClelland (1980) also described gastric ulcers in harbour seals associated with *P. decipiens* (s.l.), while Valtonen et al. (1988) found clusters of *C. osculatum* in the stomach walls of grey seals from the Baltic Sea.

The lesions observed in harbour seals resemble those reported in other marine mammals, such as harbour porpoises (*Phocoena phocoena*), where clusters of *A. simplex* (s.l.) have been associated to these lesions (Lehnert et al. 2007; Ryeng et al. 2022; Siebert et al. 2006).

The identification and reporting of these parasites from their pinniped definitive hosts provide valuable insights into their ecology and evolutionary adaptation to them. This information is important for understanding host-parasite relationships, population history, and the migration patterns of their hosts. Moreover, these findings have significant implications for public health, as anisakid nematodes reproducing with different fitness in pinnipeds have zoonotic potential through their infective third-stage larvae occurring in fish (L3). These larvae are commonly found in economically important fish species destined for human consumption, thereby affecting food safety and quality (EFSA 2024; Levsen et al. 2018; Mattiucci et al. 2018; Shamsi and Barton 2023). Given that seal stomachs can harbor large quantities of anisakid nematodes, and their larvae are frequently identified in fish species along the Norwegian coast (Berland 2003), further research is essential to enhance our understanding of parasite populations and their host specificity. While *Anisakis* spp. exhibit a broad distribution across coastal, oceanic and pelagic ecosystems, with prevalence varying significantly between broad geographic regions, anisakids maturing in pinnipeds, such as *Contracaecum* and *Phocanema*, appear to be more closely associated with the distribution of their definitive hosts and coastal environments (Cipriani et al. 2025; Gay et al. 2018; Jensen and Idås, 1992; Levsen et al. 2022; Mattiucci and Nascetti 2008).

## Conclusion

The presence of adult and mature specimens of *Anisakis simplex* (s.s.) in harbour seals, as recently reported from Danish waters (Kumas et al. 2025) and confirmed in the present study, represents a noteworthy finding. The ability of this *Anisakis* species to reach maturity in harbour seals may have been previously overlooked, or it could reflect a recently acquired capacity to develop in non-typical definitive hosts, potentially influenced by changing environmental conditions. In the Anthropocene, multiple human impacts on marine ecosystems—such as rising sea temperatures, overfishing of certain fish species, coastal industrial activity, and increased maritime traffic—are creating cascading effects that alter ecological patterns at both regional and larger scales. These changes may result in the depletion of common prey for harbor seals, forcing shifts in dietary composition or driving migration to alternative haul-out areas. Parasites, in turn, can respond with opportunistic plasticity; the ability of *A. simplex* (s.s.) to adapt to harbour seals as suitable hosts, with high fitness, as independently observed in recent times, may represent an important ecological mechanism that facilitates the maintenance and potential expansion of its life cycle in coastal and fjord ecosystems.

Although based on a limited number of individuals, this study highlights the urgent need for more systematic investigations involving a larger number of seals from multiple geographic areas investigated with genetic molecular tools. This is especially relevant considering that the most comprehensive studies from Norwegian waters date back over two decades and did not incorporate molecular genetic tools. Thus, systematic and targeted efforts are needed to clarify infection dynamics of anisakid parasites in pinniped definitive hosts and to better assess the role of these hosts in maintaining and spreading potentially zoonotic parasite species to commercial fish hosts along the Norwegian coastline.

## Data Availability

All data generated or analyzed during this study are included in this published article. http://www.ncbi.nlm.nih.gov/genbank
